# Hemocyte Responses of the Colorado Potato Beetle, *Leptinotarsa decemlineata*, and the Greater Wax Moth, *Galleria mellonella*, to the Entomopathogenic Nematodes, *Steinernema feltiae* and *Heterorhabditis bacteriophora*

**DOI:** 10.1673/031.011.7501

**Published:** 2011-06-29

**Authors:** L. Ebrahimi, G. Niknam, G. B. Dunphy

**Affiliations:** ^1^ Nematology Lab., Department of Plant Protection, Faculty of Agriculture, University of Tabriz, Tabriz, Iran; ^2^ Department of Natural Resource Sciences, McGill University, Macdonald Campus, Quebec, Canada

**Keywords:** cellular encapsulation, insect, melanization, resistant host

## Abstract

Hemocyte encapsulation reactions of infective juveniles of two Iranian isolates of the entomopathogenic nematodes, *Heterorhabditis bacteriophora* Poinar (Rhabditina: Heterorhabditidae) and *Steinernema feltiae* Filipjev (Tylenchina: Steinernematidae), were compared in the economic pest Colorado potato beetle, *Leptinotarsa decemlineata* Say (Coleoptera: Chrysomelidae), and the greater wax moth, *Galleria mellonella* L. (Lepidoptera: Pyralidae). The former was a more responsive host than the latter and the hemocyte responses occurred sooner and more extensively. Complete encapsulation of some of the nematodes occurred by 4 h post injection for *H. bacteriophora* in both *L. decemlineata* and *G. mellonella*, and by 2 h pi for *S. feltiae* in *L. decemlineata*. The percentage of encapsulation from 24 h to 72 h pi in *L. decemlineata* was 86.2% for *S. feltiae* and 39% for *H. bacteriophora*. In *G. mellonella* there were no encapsulation or melanization responses against *S. feltiae*, whereas when *H. bacteriophora* was encapsulated and melanized (16.7%) the encapsulation level was lower than in *L. decemlineata*. This study may contribute to effectively selecting entomopathogenic nematode species active against significant economic pests based on the latter's cellular immune response.

## Introduction

The entomopathogenic nematode-bacterium complexes of *Steinernema-Xenorhabdus* and *Heterorhabditis-Photorhabdus* have high potential as lethal biological control agents against many cryptic and non-cryptic insect species (Burnell and Stock 2000; Koppenhöfer 2007). The Colorado potato beetle, *Leptinotarsa decemlineata* Say (Coleoptera: Chrysomelidae), is an important worldwide potato pest (Hitchner 2007). This insect was a quarantined pest in Iran until 1984, when it reported in Ardebil province (Nouri Ganbalani 1986). The pest is killed by numerous, but not all, entomopathogenic nematode species and survival of the insect requires encapsulation of the infective juveniles of the nematodes (Thurston 1991; Thurston et al. 1994; Armer et al. 2004). *Galleria mellonella* L. (Lepidoptera: Pyralidae), the greater wax moth, is a pest of beehives; the larvae feed on wax and do considerable damage (Triplehorn and Johnson 2005) to the wax and to honey production in Iran (Goldansaz 1992).

In general, lepidopteran and coleopteran insect juvenile innate immune systems can be divided into two interactive parts (*i*) a cellular system involving hemocytes, which participated in phagocytosis, nodulation, and encapsulation; and (*ii*) a humoral system (e.g. soluble antimicrobial compounds, reactive oxygen radicals, melanin formation, and clotting) (Götz and Boman 1985; Dunphy and Thurston 1990; Feldhaar and Gross 2008). Cellular encapsulation is an insect hemocytic reaction against both living and non-living biotic objects too large for phagocytosis, and results in a multilayer cellular capsule surrounding foreign objects in the both insect orders (Götz and Boman 1985).

Despite the efficacy of many species of entomopathogenic nematodes in biological control, insects have evolved different kinds of defense mechanisms against them. Insects may avoid nematode parasitism by active resistance involving behavioral avoidance and grooming (Wang et al. 1995). The host also has passive resistance mechanisms such as the physico-chemical nature of the insect cuticle including cuticular thickness, melanizing phenoloxidase, tissue and mucus penetration, and dealing with the gut redox and enzyme content (Simões and Rosa 1996). Once past the physical barriers, the pathogens encounter the humoral and cellular defenses in the host hemolymph, the most common type of cellular response to the metazoan being encapsulation (Poinar 1979; Dunphy and Thurston 1990).

Insect hemocytic responses to steinernematids and heterorhabditids represent a continuum from effective encapsulation to no response. Effective cellular encapsulation in which different species of nematodes die commonly occurs in insects from diversified orders including the Coleoptera and Lepidoptera (Armer et al. 2004; Steiner 1996; Thurston et al. 1994; Wang et al. 1994, 95). Wang et al. (1994, 1995) demonstrated that some *S. galseri* and *Heterorhabditis bacteriophora* Poiner (Rhabditina: Heterorhabditidae) individuals, although encapsulated and melanized in hemocoel of *P*. *japonica* like the other infective juveniles, are free of hemocytes 24 h after injection because they escaped from the capsule. However, *Steinernema feltiae* Filipjev (Tylenchina: Steinernematidae) in *G. mellonella* (Brivio et al. 2002), *S*. *carpocapcae* in *G*. *mellonella* (Dunphy and Webster 1987; Walter et al. 2008) and in *Malacosoma disstria* (Walter et al. 2008) are not encapsulated because they suppress the cellular immune response of the insects. Li et al. (2007) showed that surface coat protein from *S. glaseri* is responsible for suppressing the immune response of the grub stage of the scarabaeid, *Exomala orientalis*, and that co-injection of this protein with *H. bacteriophora* into *E. orientalis* significantly reduced melanization of the normally extensively melanized nematode.

The present study documents the interaction of the two Iranian isolates of entomopathogenic nematodes *H. bacteriophora* and *S. feltiae* with an endemic economically important population of *L. decemlineata* and *G. mellonella* (an insect used often in the culturing of the entomopathogenic nematodes) as well as temporal considerations of the cellular encapsulation process in both insect species. The present combination of nematode species and host species studied in terms of hemocyte attachment to the nematodes is unique to the present study.

## Material and Methods

### Nematodes

The nematode species, *H. bacteriophora* and *S. feltiae*, were isolated from soil samples collected from East Azarbaijan province in northwest Iran using *G. mellonella* as nematode traps (Woodring and Kaya 1988). Infective juveniles of the nematodes cultured in last instar larvae of greater wax moth, *G. mellonella* (Woodring and Kaya 1988) were stored in 40 ml distilled water at 5° C and used in experiments within 30 days. Before use, the nematodes were kept at 25° C for 20–30 min for adaptation to the room temperature, during which they were disinfected by exposure to the surface disinfectant, hyamine (20% W/V H_2_O), for five min and rinsed with Ringer's solution (1% W/V NaCl) at 25° C washing by gravity three times.

### Insect colonies

Over-wintering adults of *L. decemlineata* were collected from potato fields in Bostanabaad (East Azarbaijan province) on June 2008 and used to establish a colony that has been maintained for one year prior to use as follows. The insects were kept in colorless plastic boxes (with walls covered with tissue paper) in a greenhouse [(26±2° C, 50 — 5% RH, and 16:8 (L:D) photoperiod]. Box lids were cut in the center and covered with a net cloth to supply ventilation. Potato foliage (Agria cultivar) was used as food. Eggs laid on the tissue paper or under of potato leaves transferred to wet tissue paper hatched after five days at 25° C. Larvae were reared on potato leaves until the prepupal stage, at which time they were placed on surface of sandy soil in plastic vases. When imagos emerged, they were transferred into a cage. The walls of the cage were covered with tissue paper to provide increased egg laying sites. This method was repeated from June to October. *L. decemlineata* prepupae were used in experiments because the stage forms in soil (Hitchner 2007) where they are susceptible to attack by nematodes (Klein 1990). Experiments were carried out at 26±2° C, 50 ± 5% RH, and 16:8 (L:D) photoperiod. Fourth instar larvae (based on size of head capsule) were separated from the other instars within 24 h of formation. These larvae became prepupae within three days under the described conditions. *G. mellonella* had been reared for more than 5 years on a modified artificial diet of Poinar (1975); the diet contained wheat flour (1200g), solid cubes wax (120g), yeast (300g), processed honey (600g), and glycerol 99% (500 ml). Incubation conditions were 26±2° C, 50 ± 5% RH, and 16:8 (L:D) photoperiod. The last instar larvae weighing 0.20±0.03 g were used in the experiments.

### Inoculation of nematodes and time courses of encapsulation process

Infective juveniles of each nematode species (25 infective individuals in 20µl Ringer's solution) were injected into the hemocoel of 48 insects of both insect species using a tuberculinin syringe. The dosage was based on the range of infective juveniles of either species used by Thurston et al. (1994) and produced discernible mortality in the present study. The prepupae of *L. decemlineata* were injected through the dorsal cuticle, after the pronotum, and *G. mellonella* via the base of the last abdominal prolegs. Ten insects of each species injected with 20 µl Ringer's solution served as the control. Insects were incubated in 9 cm in diameter glass Petri dishes. Because of the non-feeding behavior of prepupae, food supplement was not applied during the experiment. Four insects were dissected at selected times after injection. The stages of encapsulation were divided into following categories: (1) one or more hemocytes attached, (2) multiple hemocyte with layer formation, (3) complete hemocytic capsule formation with normal round hemocytes associated with the outer most layer, (4) partial melanization initiated, and (5) complete melanization. The numbers of encapsulated nematodes in the hemocoel and fat body as well as non-encapsulated nematodes were counted under a LEICA MZ 125 stereomicroscope (www.leica-microsystems.com). Mortality of insects was recorded when it occurred. Digital images of different stages of hemocyte attachment were recorded using an OLYMPUS BX41 microscope (www.soft-imaging.net) provided with a DP50 camera. Experiments were replicated twice.

### Statistics

The percentage of encapsulated nematodes was based on the number of juveniles completely encased in hemocytes at least one cell layer thick compared to the total number of recovered nematodes. Encapsulation percentages were transformed by arcsin√p, where p is the decimal value of the percentage, and presented in the corresponding tables (Sokal and Rohlf 1969), to allow statistical analysis by analysis of variance and Ducan's multiple-range test using SAS software (SAS Institute 2004) in which normal data distribution was determined within the program. A significance level of α=0.05 was used. All graphic data were presented as the non-transformed data (Sokal and Rohlf 1969).

## Results

### Hemocyte attachment to *Heterorhabditis bacteriophora Leptinotarsa decemlineata*

**Figure 1.**
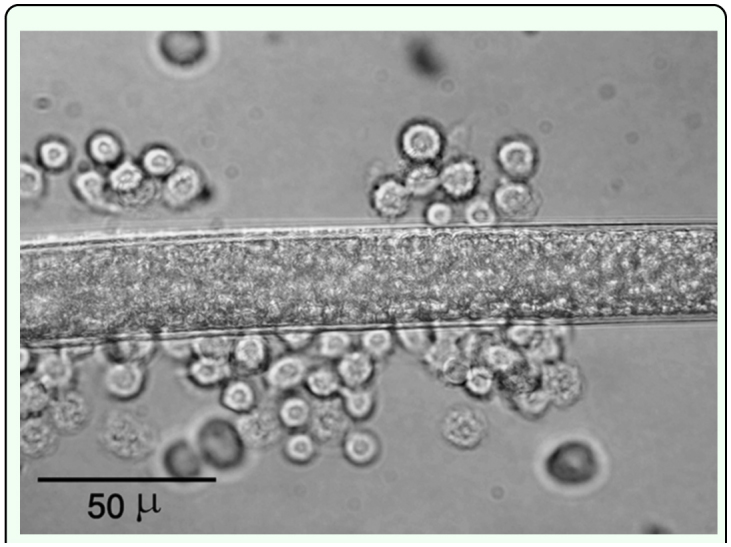
Attachment of hemocytes of *Leptinotarsa decemlineata* to the surface of *Heterorhabditis bacteriophora*, 45 min pi. High quality figures are available online.

**Figure 2.**
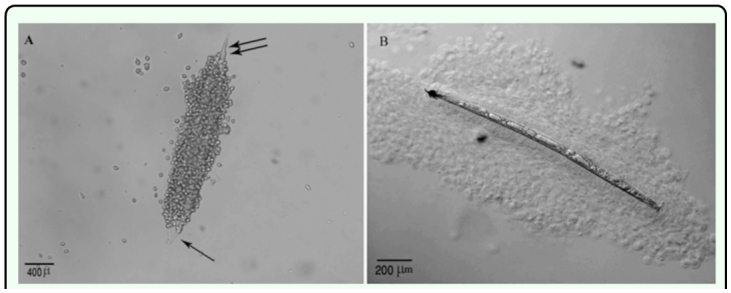
*Heterorhabditis bacteriophora* in *Leptinotarsa decemlineata*, (A) During the initial four hours after injection the head (single arrows) and tail (double arrows) were free of hemocytes. (Bright field), (B) Complete encapsulation occurred by 16 h post-injection. [Nomarski Differential Interference Contrast Observation (DIC)]. High quality figures are available online.

**Figure 3.**
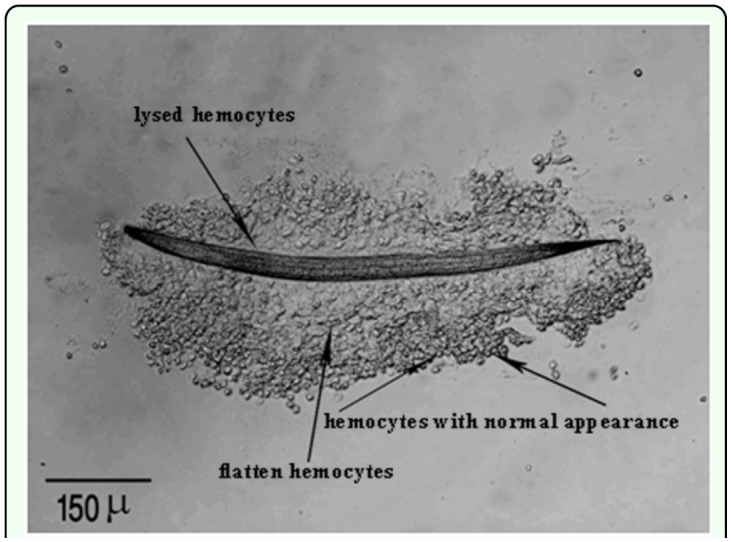
Complete capsule around *Heterorhabditis bacteriophora* in *Leptinotarsa decemlineata*, consists of three layers 24 h pi (DIC). High quality figures are available online.

In all cases, infective juveniles were encapsulated effectively preventing their development into male and female adults. However, where juveniles developed there was no encapsulation of the adults of either sex. Attachment of hemocytes to *H. bacteriophora* started within 15 min after injection (Figure 1, Table 1). During the initial 15 min to 4 h after injection the hemocytes loosely adhered to the nematode surface except for the anterior and caudal ends (Figure 2A), and separated when the nematode moved. By 16 h after injection hemocytes tightly surrounded the entire nematode (Figure 2B). Melanization of the capsule was initiated in the innermost layers of lysing hemocytes, near the surface of nematode's cuticle, with the tail and head regions being the first parts melanized (Figure 2B) followed by middle region. Three-layered capsules surrounded the nematodes by 24 h after injection. In the innermost layer hemocytes were completely flattened and compacted, hemocytes were approximately normal in appearance in middle layers of the capsule, while hemocytes in the outermost layers showed a normal appearance (Figure 3) possibly representing detaching or attaching cells. Encapsulated and melanized nematodes were alive 48 h after injection. Insect mortality due to *H. bacteriophora* infection was recorded 100% at 72 h after injection, and symbiotic bacteria were observable by this time. Control insects were alive at 72 h after injection.

**Table 1.**
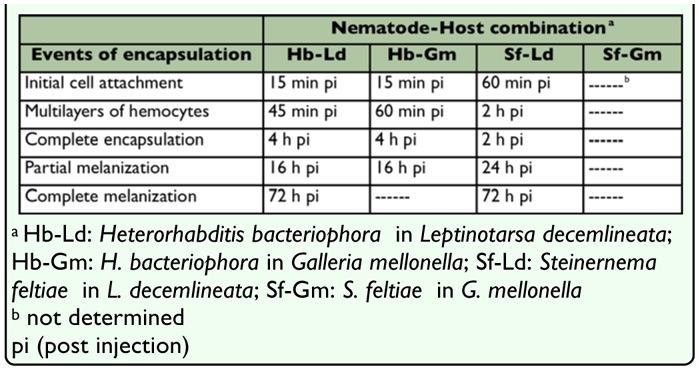
Encapsulation events around *Heterorhabditis bacteriophora* and *Steinernema feltiae* in two host species, *Leptinotarsa decemlineata* and *Galleria mellonella*.

**Figure 4.**
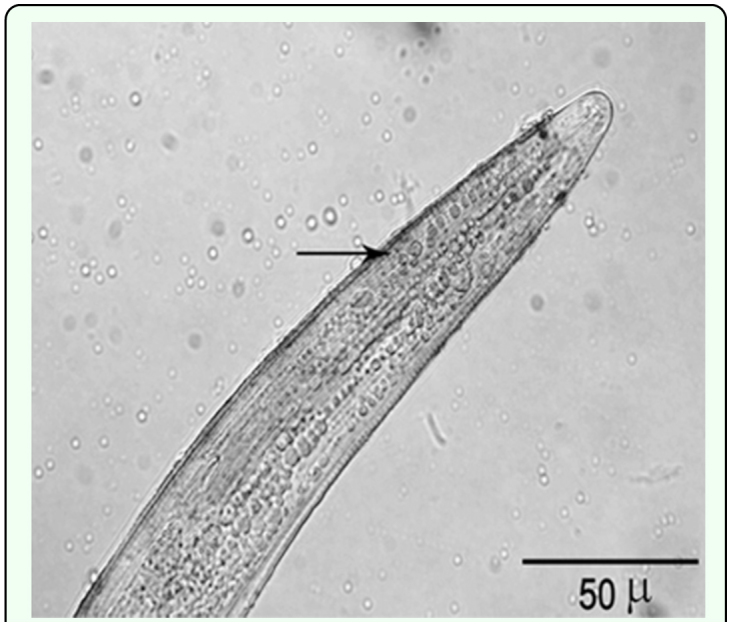
Live *Heterorhabditis* *bacteriophora* with a thin brown layer (arrow) on the cuticle surface without hemocytes (DIC). High quality figures are available online.

From 24 h to 72 h after injection 39% (Table 2, P < 0.05, n = 8 larvae) of the nematodes were fully encapsulated in the hemocoel, and approximately 7.1% (P < 0.05, n = 8 larvae) of the nematodes in the fat body were without visible hemocytes or melanization and were active whereas still fewer (Table 2, P > 0.05, n = 8 larvae) were also alive, but coated in thin brown layer without a cellular capsule (Figure 4).

**Figure 5.**
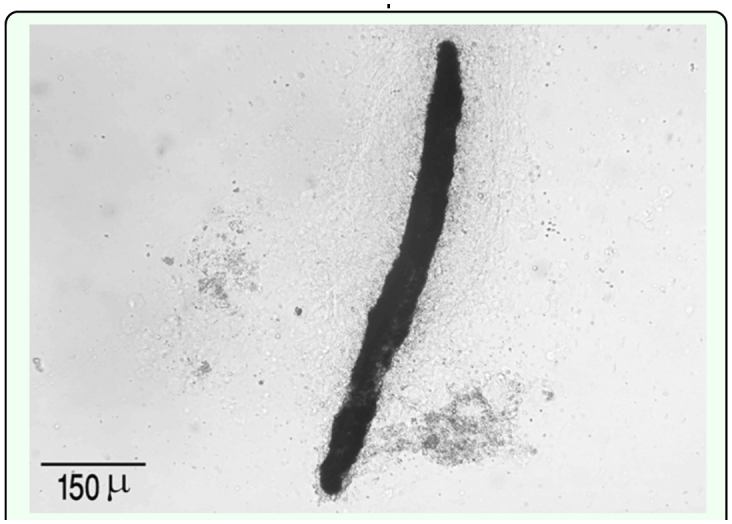
Completely melanized capsule around *Heterorhabditis bacteriophora* in *Leptinotarsa decemlineata* 72 h pi in hemocoel (DIC). High quality figures are available online.

By 72 h after injection completely melanized nematodes were observed (Figure 5), however fewer nematodes (4.2%, P < 0.05, n = 8 larvae) were encapsulated and melanized inside the fat body. Some nematodes appeared to have embedded within fat body cells lacking hemocyte involvement.

**Table 2.**
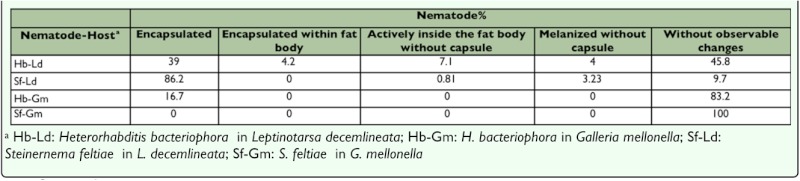
Percentage of encapsulation from 24 to 72 h pi in different nematode-host combinations.

### Galleria mellonella

**Figure 6.**
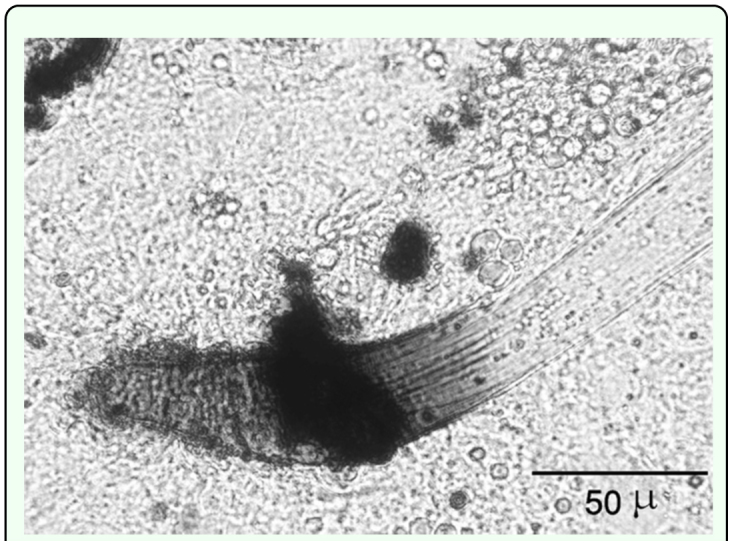
Partial melanization in anterior region of *Heterorhabditis bacteriophora* 16 h pi in the hemocoel of *Galleria mellonella* larvae (DIC). High quality figures are available online.

As summarized in Table 1, loose hemocytes attached to the surface of *H. bacteriophora* were observed within 15 min after injection. After 4 h after injection a multilayered hemocyte capsule completely surrounded the nematodes. Partial melanization in anterior region of the nematodes occurred by 16 h after injection (Figure 6). *Photorhabdus* sp. was released before 16 h after injection since the bacteria were observable in insect hemocoel at 16 h after injection. Moreover, the 83.2% of insect mortality that occurred 16 h after injection may be due to symbiotic bacteria activity. At 24 h after injection significantly fewer nematodes were encapsulated compared with those in *L. decemlineata* (Table 2, P > 0.05, n = 8), and of encapsulated nematodes most were alive and mobile. By 32 h after injection, 100% of the treated *G. mellonella* larvae were found dead while control larvae were alive and bacteria-free. At 24 h capsules were loose and essentially all of the encapsulated nematodes had escaped. There was no discernible invasion of the fat body by the nematodes (Table 2).

### Hemocyte attachment to *Steinernema feltiae Leptinotarsa decemlineata*

**Figure 7.**
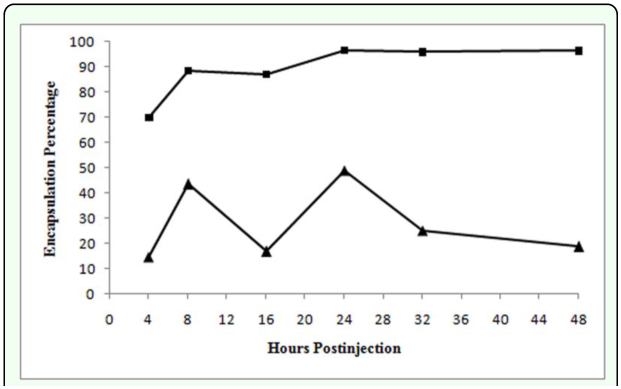
Percentage of hemocyte attachment due to capsule formation during different time courses around *Heterorhabditis bacteriophora* (solid triangles) and *Steinernema feltiae* in *Leptinotarsa decemlineata* (solid square). High quality figures are available online.

Only infective juveniles were extensively encapsulated, the extent during the later stages of the pathology being about twice that of *H. bacteriophora* (Table 2, P > 0.05, n = 8 larvae), no encapsulated mature male and female nematodes were observed. It should be noted that the encapsulation percentages of *S. feltiae* and *H. bacteriophora* were not similar in either magnitude or kinetics (Table 2, Figure 7) reflecting differences in nematode physical and active interaction with the defenses of the host. Unlike *H. bacteriophora* in *L. decemlineata*, hemocytes did not adhere to the nematodes during the initial 45 min after injection, however, attachment occurred around the middle and head region of the nematodes by 60 min after injection (Table 1). Multiple layers of hemocytes were detected by 2 h after injection. During 16 h after injection the number of surrounding layers of hemocytes increased as the inner layers compacted. Similar to *H. bacteriophora*, both ends of the nematodes were the last parts to which hemocytes adhered and the first part exhibiting melanization. Here melanization was detected much later than for *H. bacteriophora* (Table 1). Brown color masses of melanin-like materials in some regions on the surface of a few nematodes without cellular surrounding capsule, were observed 24 h after injection (Table 2). However, by 72 h after injection the entire surface of the cuticle was covered with a thick layer of melanin. Encapsulated nematodes were alive at 48 h after injection. Insect mortality was 50% at 72 h after injection and symbiotic bacteria were also observed. No mortality occurred in control larvae.

### Galleria mellonella

In *G. mellonella* larvae neither hemocytes attachment nor melanization occurred on the nematodes (Table 1). Insect mortality initially occurred 16 and 24 h after injection and by 24 h after injection 100% were dead. At 16 h after injection *Xenorhabdus* sp. were observed in hemolymph in high density. Histolysis of the insect body tissues and the fat body, but not the digestive system, was observed at this time.

## Discussion

The three-layered capsule surrounding both nematode species in *L. decemlineata* by 24 h after injection was similar to results in other lepidopteran species (Götz and Boman 1985; Pech and Strand 2000) with the capsules being composed of completely flattened and compacted hemocytes in the innermost layer, hemocytes with approximately normal appearance in the middle layers of the capsule and finally, hemocytes with a normal appearance in the outermost layers. The latter may represent either attaching or detaching.

These results showed that both rate and details of nematode encapsulation varied with the host and nematode species. Weak hemocyte reaction to nematodes in *G. mellonella*, that has been commonly reported in the literature (Milstead 1979; Dunphy and Thurston 1990), allows the release of the bacteria into the hemocoel ensuring the survival of the nematodes (Cox-Foster et al. 2002). Encapsulation of *Steinernema* sp. and *Heterorhabditis* sp. are reported in *L. decemlineata* (Dunphy and Thurston 1990; Thurston 1991; Armer et al. 2004). However, Armer et al. (2004) described encapsulation of *H. marelatus* by *L. decemlineata* in vivo and in vitro in hanging drops of hemolymph and in *G. mellonella*. The results of the present study indicate that encapsulation of *H. bacteriophora* in *L. decemlineata* and *G. mellonella* occurs by 2 h after injection; however Dunphy and Thurston (1990) showed no evidence of encapsulation of *H. bacteriophora* in these host species up to 4 h after injection. Thurston (1991) and Thurston et al. (1994) found that cellular encapsulation of *S. carpocapsae* occurs in *L. decemlineata*, and the adhering hemocytes were observed 1.5 hours after injection with a complete multilayered capsule by 4 h after injection; such encapsulated nematodes appeared dead. It was observed, however, that encapsulated *H. bacteriophora* with melanized capsules and encapsulated *S. feltiae* were alive and active at 48 h after injection. Such differences may be due to using different nematode and insect strains and culture conditions. It seems that some of the infective juveniles that maintain their second cuticle [(reflecting the effects of rearing and storage conditions (Campbell and Gaugler 1991)] were alive inside the melanized capsule until 48 h after injection. It is unlikely that the ensheathed nematodes reflect an experimental artifact since *H. bacteriophora* retains sheaths (Liu and Glazer 2000), and thus would be expected to encounter hemocytes. Also, although steinernematid exsheathment and desheathment readily occurs favoring host infection (Campbell and Gaugler 1991), penetrating infective juveniles lacking sheaths are known (Ishibashi and Takii 1993); thus, secondary *S*. *feltiae* cuticle interaction with hemocytes is expected. That *S. feltiae* infective individuals with and without sheaths were encapsulated, was expected since the sheath does not confer desiccation resistance (Menti et al. 1997) or offset osmotic-induced chemical stress (Qui et al. 2000) implying chemical similarities of the epicuticle of both the secondary cuticle and cuticle, and this is supported by the similarities in ultrastructure (Menti et al. 1997). This may apply to the cuticles of *H. bacteriophora*. The sheath protected the nematodes, but not by reducing the availability of hemocytes, since the nematodes encapsulated even after escape from the capsule. This also suggests that, unlike Campbell and Gaugler's (1991) findings in which chemical disinfectant alters cuticle composition, the use of hyamine does not affect the cuticle sufficiently to influence hemocyte responses. Factors such as insect's nutrition and growth parameters such as temperature, photoperiod, and relative humidity (Moret 2006) and importantly, the nematode strain and bacterial species/strain (Bedding et al. 1983), may affect host immune physiology. Different interactions of the bacterial, nematode, and host genotypes are also responsible for differences in host cellular immune response to nematodes (Li et al. 2007). The ‘infective juvenile’ is the survival stage of the nematodes having morphological and physiological adaptations to remain in the environment without nourishment (Poinar 1990). It is possible that the nutritional and waste removal barriers imposed by capsules would be less effective against this stage of nematodes.

Steiner (1996) described encapsulation and melanization of *S. feltiae* and *S. kraussei* in larvae of the black vine weevil, *Otiorhynchus sulcatus*, in which encapsulation was started around the anus and/or head of the nematodes. However, our results show that the posterior and anterior region of the nematodes were the last points that were encapsulated. It has been reported that *Xenorhabdus* of *S. carpocapsae* is released by esophageal pumping down the intestine and out the anus (Snyder et al. 2007); the encapsulation reported herein may ensure bacterial release even as encapsulation occurs. The bacteria are thus free to produce a myriad of hemocyte toxic components including enzymes. In addition, the hemocytes initially adhere around esophageal region, implying that the secretory-excretory pore exudates may trigger initial hemocytes attachment or alternatively a biochemical component of the cuticle in the region is involved. Its significance remains unknown. In *O. sulcatus*, partially melanized nematodes were found alive (Steiner 1996) as they were in the present study for *H. bacteriophora* in *L. decemlineata*. Attachment of *G. mellonella* hemocytes to *H. bacteriophora* occurs within 15 min after injection, but by 24 h capsules are loose, which may explain also the existence of the capsules without nematodes. Symbiotic bacteria damage the host insect hemocytes (Dunphy and Hulbert 1995; da Silva et al. 2000), diminishing hemocytes adhesion, and thus nematode escape from the damaged capsules may be partially attributed to bacterial activity. These observations are supported by the results of Li et al. (2007) who showed that blood cells of *G. mellonella* as a susceptible host recognized *H. bacteriophora* at 1 and 24 h after injection, but a significant percentage of the nematodes escaped from attached hemocytes.

In this study, *S. feltiae* did not trigger cellular encapsulation in *G. mellonella*. The insect's cellular immune system was not able to recognize the nematode as non-self, possibly as described by Brivio et al. (2002), in which host lipopolysaccharide-like binding proteins masked the pathogen and suppressed phenoloxidase - both contributing to limiting capsule formation. They indicated also that in *G. mellonella*, larvae of *S. feltiae* induce a speedy suppression of phenoloxidase activity, thus avoiding host humoral encapsulation. Dunphy and Webster (1987) and Walter et al. (2008) stated that *S. carpocapsae* avoids encapsulation in *G. mellonella* by two mechanisms, the immediate one being the lipid nature of the nematode cuticle (Dunphy and Webster 1987) and the other being hydrophilic exudates of live nematodes that limit hemocytes adhesion (Walter et al. 2008).

Encapsulation percentage of *S. feltiae* increased rapidly during initial interaction in *L. decemlineata* but more slowly thereafter, whereas encapsulation fluctuated extensively for the few *H. bacteriophora* encapsulated by this insect species. For the former pathogen this may indicate that not all the nematodes were encapsulated for reasons yet to be determined. The decrease of encapsulation by the latter nematode species can be related to both sporadic release of bacterial endotoxin and the enzymatic activity of symbiotic bacteria that damage the hemocytes (Dunphy and Hulbert 1995; da Silva et al. 2000). The total number of damaged hemocytes in both *G. mellonella* and *M. disstria* larvae increased up to 24 h after injection when injected with live axenic *S. carpocapsae* reflecting nematode exudates (Walter et al. 2008). Götz and Boman (1985) demonstrated that the cell number (and the volume) of a capsule has its peak 12–24 h after introduction of the foreign object and decreases during the following days. Results of the present study showed that hemocytes' adhesion to the *H. bacteriophora*, but not *S. feltiae* in *L. decemlineata*, decreased after 24 h after injection by which time the maximum encapsulation percentage was observed. Probably, apoptosis induced by bacterial infection could be responsible for hemocytes' death (Cho and Kim 2004).

Entomatopathogenic nematodes kill their host in a variety of ways, including releasing the bacteria before complete encapsulation (Wang et al. 1995), avoidance of the hemocytic immune response of *L. decemlineata* by entering into the fat body as described for *H. bacteriophora* in *G. mellonella* (Milstead 1979), leaving the encapsulated cuticle, and escaping from the fat body (Wang et al. 1994, 1995) possibly after acquiring self-antigens. Results of this study indicate that cellular encapsulation of nematodes in *L*. *decemlineata* at the dosage used is not an effective defensive mechanism, and despite substantial encapsulation of the nematodes, insect mortality occurred for *S. feltiae* and *H. bacteriophora* injections 72 h after injection. Thurston et al. (1994) demonstrated that cellular encapsulation of *S. carpocapsae* and insect mortality were dose-dependent, and encapsulation was decreased by increasing nematode loading. According to the Cox-Foster et al. (2002), *L. decemlineata* is a semipermissive host for both *H. bacteriophora* and *S. feltiae* due to the time-consuming process of encapsulation; nematodes have enough time to release their symbiotic bacterium.

## References

[bibr01] Armer CA, Rao S, Berry RE (2004). Insect cellular and chemical limitations to pathogen development: the Colorado potato beetle, the nematode *Heterorhabditis marelatus*, and its symbiotic bacteria.. *Journal of Invertebrate Pathology*.

[bibr02] Bedding RA, Molyneuxand AS, Akhurst RJ (1983). *Heterorhabditis* spp., *Neoaplectana* spp., and *Steinernema kraussei:* Interspecific and intraspecific differences in infectivity for insects.. *Experimental Parasitology*.

[bibr03] Brivio MF, Pagani M, Restelli S (2002). Immune suppression of *Galleria mellonella* (Insecta, Lepidoptera) humoral defenses induced by *Steinernema feltiae* (Nematoda, Rhabditida): involvement of the parasite cuticle.. *Experimental Parasitology*.

[bibr04] Burnell AM, Stock SP (2000). *Heterorhabditis, Steinernema* and their bacterial symbiontslethal pathogens of insects.. *Nematology*.

[bibr05] Campbell LR, Gaugler R (1991). Mechanisms for exseathment of entomopathogenic nematodes.. *International Journal of Parasitology*.

[bibr06] Cho S, Kim Y (2004). Hemocyte apoptosis induced by entomopathogenic bacteria, *Xhenorhabdus* and *Photorhabdus*, in *Bombyx mori*.. *Journal of Asia Pacific Entomology*.

[bibr07] Cox-Foster DL, Kazi A, Miller K (2002). Abstracts of the fourth international symposium on molecular insects science.. *Journal of Insect Science*.

[bibr08] da Silva CCA, Dunphy GB, Rau ME (2000). Interaction of *Xenorhabdus nematophilus* (Enterobacteriaceae) with the antimicrobial defenses of the house cricket, *Acheta domestica*.. *Journal of Invertebrate Pathology*.

[bibr09] Dunphy GB, Hulbert RE (1995). Interaction of avirulent transpositional mutants of *Xenorhabdus nematophilus* ATCC 19061 (Enterobacteriaceae) with the antibacterial systems of non-immune *Galleria mellonella* (Insecta) larvae.. *Journal of General and Applied Microbiology*.

[bibr10] Dunphy GB, Thurston S, Gaugler R, Kaya HK (1990). Insect immunity.. *Entomopathogenic Nematodes in Biological Control*.

[bibr11] Dunphy GB, Webster JM (1986). Influence of the Mexican strain of *Steinernema feltiae* and its associated bacterium *Xenorhabdus nematophilus* on *Galleria mellonella*.. *Journal of Parasitology*.

[bibr12] Dunphy GB, Webster JM (1987). Partially characterized components of the epicuticle of dauer juvenile *Steinernema feltiae* and their influence on hemocyte activity in *Galleria mellonella*.. *Journal of Parasitology*.

[bibr13] Feldhaar H, Gross R (2008). Immune reactions of insects on bacterial pathogens and mutualists.. *Microbes and Infection*.

[bibr14] Goldansaz SH (1992). *An investigation on the biological aspects of greater and lesser wax moth, Galleria mellonella and Achroia grisella, under controlled storage and laboratory conditions*..

[bibr15] Götz P, Boman HG, Kerkut GA, Gilbert LI (1985). Insect immunity.. *Comprehensive insect physiology, biochemistry and pharmacology*.

[bibr16] Hitchner EM (2007). *Investigations of the integrated pest management of Colorado potato beetle, Leptinotarsa decemlineata (Say): Host plant preference, development of semiochemical-based strategies, and evaluation of a novel insecticide*..

[bibr17] Ishibashi N, Takii S (1993). Effects of insecticides on movement, nictation, and infectivity of *Steinernema carpocapsae*.. *Journal of Nematology*.

[bibr18] Klein GM, Gaugler R., Kaya H. K. (1990). Efficacy against soil-inhabiting insect pests.. *Entomopathogenic nematodes in biological control*..

[bibr19] Koppenhöfer AM, Lacey LA, Kaya HK (2007). Nematodes.. *Field manual of techniques in invertebrate pathology application and evaluation of pathogens for control of insects and other invertebrate pests*.

[bibr20] Li XY, Cowles RS, Cowles EA, Gaugler R, Cox-Foster DL (2007). Relationship between the successful infection by entomopathogenic nematodes and the host immune response.. *International Journal for Parasitology*.

[bibr21] Liu QZ, Glazer I (2000). Desiccation survival of entomopathogenic nematodes of the genus *Heterorhabditis*.. *Phytoparasitica*.

[bibr22] Menti H, Wright DJ, Perry RN (1997). Desiccation survival of populations of the entomopathogenic nematodes *Steinernema feltiae* and *Heterorhabditis* from Greece and the UK.. *Journal of Helminthology*.

[bibr23] Milstead JE (1979). Pathophysiological influences of *Heterorhabditis bacteriophora* complex on seventh-instar larvae of the greater wax moth, *Galleria mellonella*: changes in the hemolymph refractive index.. *Journal of Invertebrate Pathology*.

[bibr24] Moret Y (2006). Trans-generational immune priming: specific enhancement of the antimicrobial immune response in the mealworm beetle, *Tenebrio molitor*.. *Proceedings of the Royal Society B: Biological Sciences*.

[bibr25] Nouri Ganbalani G (1986). *Colorado potato beetle* (in Persian)..

[bibr26] Patel MN, Wright DJ (1998). The ultrastructure of the cuticle and sheath of infective juveniles of the entomopathogenic steinernematid nematodes.. *Journal of Helminthology*.

[bibr27] Pech LL, Strand MR (2000). Plasmatocytes from the moth *Pseudoplusia includens* induce apoptosis of granular cells.. *Journal of Insect Physiology*.

[bibr28] Poinar GO (1975). *Entomogenous Nematodes*..

[bibr29] Poinar GO (1979). *Nematodes for biological control of insects*..

[bibr30] Poinar GO, Gaugler R, Kaya HK (1990). Biology and taxonomy of Steinernematidae and Heterorhabditidae.. *Entomopathogenic nematodes in biological control*.

[bibr31] Qiu L, Lacey MJ, Bedding RA (2000). Permeability of infective juveniles of *Stenernema carpocapsae* to glycerol during osmotic dehydration and its effect on biochemical adaptation and energy metabolism.. *Comparative Biochemistry and Physiology Part*.

[bibr32] SAS Institute (2004). *SAS Enterprise Guide ver. 3.0*..

[bibr33] Simões N, Rosa JS (1996). Pathogenicity and host specificity of entomopathogenic nematodes.. *Biocontrol Science and Technology*.

[bibr34] Snyder H, Stock SP, Kim SK, Flores-Lara Y, Forst S (2007). New insights into the colonization and release processes of *Xenorhabdus nematophila* and morphology and ultrastructure of the bacterial receptacle of its nematode host, *Steinernema carpocapsae*.. *Applied and Environmental Microbiology*.

[bibr35] Sokal RR, Rohlf FJ (1969). *Biomerty*..

[bibr36] Steiner WA (1996). Melanization of *Steinernema feltiae* Filipjev and *S. kraussei* Steiner in larvae of *Otiorhynchus sulcatus* (F.).. *Fundamental and Applied Nematology*.

[bibr37] Thurston GS (1991). *The physiological ecology of Steinernema carpocapsae (Nematoda: Rhabditida) as it relates to efficacy in controlling soil- inhabiting insect pests*..

[bibr38] Thurston GS, Yule WN, Dunphy GB (1994). Explanations for the low susceptibility of *Leptinotarsa decemlineata* to *Steinernema carpocapsae*.. *Biological Control*.

[bibr39] Triplehorn CA, Johnson NF (2005). *Borror and Delong's Introduction to the Study of Insects*.

[bibr40] Walter NT, Dunphy GB, Mandato CA (2008). *Steinernema carpocapsae* DD136: metabolites limit the non-self adhesion responses of hemocytes of two lepidopteran larvae, *Galleria mellonella* (F. Pyralidae) and *Malacosoma disstria* (F. Lasiocampidae).. *Experimental Parasitology*.

[bibr41] Wang Y, Gaugler R, Cui L (1994). Variations in immune response of *Popillia japonica* and *Acheta domesticus* to *Heterorhabditis bacteriophora* and *Steinernema* species.. *Journal of Nematology*.

[bibr42] Wang Y, Campbell JF, Gaugler R (1995). Infection of entomopathogenic nematode *Steinernema glaseri* and *Heterorhabditis bacteriophora* against *Popillia japonica* (Coleoptera, Scarabaeidae) larvae.. *Journal of Invertebrate Pathology*.

[bibr43] Woodring JL, Kaya HK (1988). *Steinernematid and heterorhabditid nematodes: a handbook of biology and techniques*..

